# Reactive Gliosis in Neonatal Disorders: Friend or Foe for Neuroregeneration?

**DOI:** 10.3390/cells13020131

**Published:** 2024-01-11

**Authors:** Justyna Gargas, Justyna Janowska, Paulina Gebala, Weronika Maksymiuk, Joanna Sypecka

**Affiliations:** NeuroRepair Department, Mossakowski Medical Research Institute, Polish Academy of Sciences, A. Pawinskiego 5, 02-106 Warsaw, Poland; jgargas@imdik.pan.pl (J.G.); jjanowska@imdik.pan.pl (J.J.);

**Keywords:** gliogenesis, neonatal astrocytes, astrogliosis, neonatal disorders, secretome, cell interactions, therapeutic strategies

## Abstract

A developing nervous system is particularly vulnerable to the influence of pathophysiological clues and injuries in the perinatal period. Astrocytes are among the first cells that react to insults against the nervous tissue, the presence of pathogens, misbalance of local tissue homeostasis, and a lack of oxygen and trophic support. Under this background, it remains uncertain if induced astrocyte activation, recognized as astrogliosis, is a friend or foe for progressing neonatal neurodevelopment. Likewise, the state of astrocyte reactivity is considered one of the key factors discriminating between either the initiation of endogenous reparative mechanisms compensating for aberrations in the structures and functions of nervous tissue or the triggering of neurodegeneration. The responses of activated cells are modulated by neighboring neural cells, which exhibit broad immunomodulatory and pro-regenerative properties by secreting a plethora of active compounds (including interleukins and chemokines, neurotrophins, reactive oxygen species, nitric oxide synthase and complement components), which are engaged in cell crosstalk in a paracrine manner. As the developing nervous system is extremely sensitive to the influence of signaling molecules, even subtle changes in the composition or concentration of the cellular secretome can have significant effects on the developing neonatal brain. Thus, modulating the activity of other types of cells and their interactions with overreactive astrocytes might be a promising strategy for controlling neonatal astrogliosis.

## 1. Introduction

Astrocytes, the predominant type of glial cells in the central nervous tissue, play diversified crucial roles during neurodevelopment and physiological central nervous system (CNS) functions during one’s lifespan. Astrocytes support neurons, providing metabolic substrates (including an astrocyte–neuron lactate shuttle to fuel the energy needs of neurons) and trophic factors [1,2,3]. During both neurodevelopment (which is intensely ongoing from about the 23rd week of embryonic life in humans) and CNS functioning throughout one’s lifespan, astrocytes are engaged in synapse formation and plasticity; due to their ability to release gliotransmitters (glutamate, D-serine, and adenosine triphosphate-ATP), astrocytes participate in intercellular communication [4,5,6,7]. Their end-feet participate in the formation and maintenance of the blood–brain barrier (BBB), which is a highly selective semipermeable border between the capillaries and the brain parenchyma that helps protect the brain from circulating neurotoxic plasma components and pathogens [8,9]. Astrocytes are also well recognized as the primary maintainers of local tissue homeostasis by regulating ion concentrations and cell metabolite concentrations [10]. Astrocytes are known for their rapid responses to homeostasis imbalance, different injuries to the CNS, and the occurrence of various pathophysiological conditions. The endogenous mechanisms initiated in response to injuries or pathophysiological clues and the extent of cell reactivity are usually the crucial discriminating factors between fostering natural reparative processes in the nervous tissue and triggering neurodegeneration. Thus, strategies aimed at governing reactive gliosis, especially during the neonatal period and in early childhood when potential compensative processes are the most effective, can yield the desired therapeutic outcomes aimed at restoring the structures and functions of nervous tissue.

## 2. Astrocytes in Neurodevelopment

**Figure 1:**
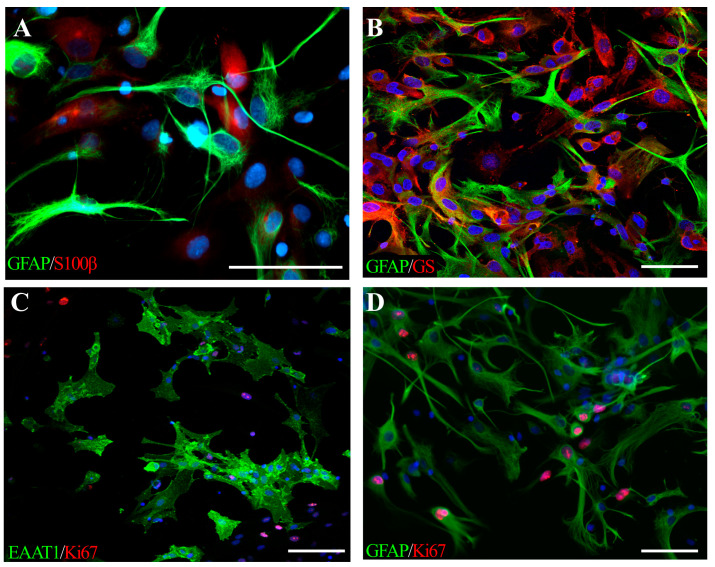
Neonatal rat astrocytes cultured in physiological normoxia (described in detail in [24]), expressing classical lineage-specific markers: (**A**) GFAP (green) and S100β (red); (**B**) GFAP (green) and GS (red), (**C**) EAAT1 (green) and Ki67 (red), a marker of dividing cells, (**D**) GFAP-positive astrocytes (green), proliferating (Ki67, red) in response to temporal oxygen-glucose deprivation. Cell nuclei are stained with Hoechst (blue). The scale bar corresponds to 50 μm.

During embryogenesis, astrocytes originate from pallial and subpallial radial glial cells (RGCs), which are considered the primary multipotential neural precursors derived from neuroepithelial cells lining the ventricles of the neural tube [11,12,13]. Neural precursor cells give rise to neurons and glial cells, including astrocytes and oligodendrocytes, which are recognized as macroglia. Neurogenesis begins in the embryonic stage and precedes gliogenesis, which is most intense in the perinatal and early postnatal periods. The switch to a gliogenic fate in the late embryonic stage is regulated by a changing gradient of patterning molecules including fibroblast growth factor 8 (FGF8), fibroblast growth factor 7 (FGF7), transforming growth factor-α (TGF-α), bone marrow protein 4 (BMP4), Wnt signaling molecules, and sonic hedgehog (Shh) in the brain, as well as BMP4 and Shh in the spinal cord [14,15]. BMP acts as an inducer of the expression of inhibitory factors, including DNA-binding protein inhibitors 1 and 2 (Id1 and Id3), and a homologue of hairy and enhancer of split 5 (Hes5), a helix-loop-helix (HLH) protein that represses genes engaged in neurogenesis. In late gestation, the JAK–STAT signaling pathway is activated by interleukin 6 (IL-6)-family cytokines, including leukemia inhibitory factor (LIF), ciliary neurotrophic factor (CNTF), and cardiotrophin-1 (CT-1) [16]. CT-1 coupling through glycoprotein gp130 and leukemia inhibitory factor receptor β (LIFRβ) receptors enhances signal transducer and activator of transcription 3 (STAT3) phosphorylation and its binding to transcriptional coactivator p300/CBP (acting as histone acetyltransferase) to activate the two major gliogenic genes: GFAP (glial fibrillary acidic protein) and S100β (calcium-binding protein β) [17]. These morphogenic proteins are among the major, but not the only, stimuli governing the transition from a neurogenic to gliogenic fate for neural precursors. Under the stimulation of a changing gradient of external stimuli, glial precursors migrate and populate the developing CNS [18,19,20]. Radial glia are considered to serve as scaffolds for cells translocating to their final destination. Glial precursors constitute a heterogeneous cell population that differs at the morphological, molecular, and functional levels in relation to the brain regions and external stimuli present in the local tissue microenvironment [21,22]. In general, highly branched protoplasmic astrocytes are prevalent in the grey matter, while fibrous astrocytes characterized by fewer and longer branches are usually found in the white matter of the CNS. Recent studies have shown that primary rat neonatal astrocytes cultured in vitro in physiologically normoxic conditions (i.e., 5% oxygen concentration, which is recognized as typical for the brain) and serum-free medium, express classical astrocytic markers such as GFAP, GS (glutamine synthetase), and EAAT1 (ex-citatory amino acid transporter 1) (Figure 1). Moreover, culturing neonatal astrocytes on the surfaces coated with the selected extracellular matrix components (like fibronectin and laminin) revealed that the expression of those markers is regulated by the contact with the applied biomimetic factors, which could be associated with the final astrocytic heterogeneity. Likewise, fibronectin was shown to stimulate the expression of GFAP and GS, both in control astrocytes and those subjected to temporary deprivation of oxygen and glucose [23].

The most intense period of astrogenesis peaks during the late embryogenesis and early postnatal periods [24,25]. During ontogenesis, astrocyte-derived active compounds regulate several processes, including upregulation of density and branching of cortical blood vessels, thus contributing to the development of intricate tissue cytoarchitecture [26,27]. In this way, astrocytes participate in the formation of a functional BBB composed of cerebral microvessels surrounded by the astrocyte end-feet. This barrier controls parenchymal homeostasis and provides the brain with oxygen and metabolic substrates while protecting it against toxins and pathogens [28,29]. Although barrier genesis begins before the onset of astrogenesis [30], those glial cells are engaged in BBB formation and maintenance, both during CNS development and in adulthood. Initially, pro-angiogenic factors like VEGF (Vascular Endothelial Growth Factor), CXCL4 (platelet factor 4), factors of Wnt/β-catenin signaling pathways, and retinoic acid are released by radial glia/neural precursor cells [31,32]. The emerging astrocytes, however, are able to potently secrete VEGF, angiopoietin-1 (Ang-1) and angiopoietin-2 (Ang-2), heme oxygenase-1 (HO-1), and endothelin-1 (ET-1) in the developing CNS [33]. Gain-of-function and loss-of-function experiments in animal models confirmed that astrocyte-derived VEGF plays an important role in the formation and growth of new blood vessels [34].

Fibronectin, an extracellular matrix (ECM) component, is another proangiogenic factor secreted by astrocytes, which has a beneficial effect on endothelial cell survival and proliferation through α5β1 and αvβ3 integrins via the mitogen-activated protein (MAP) kinase signaling pathway [35]. As an ECM constituent, fibronectin also serves as a scaffold for endothelial cell migration and vascularization [36]. Other factors of ECM secreted by astrocytes include laminins, which are glycoproteins localized in the basement membrane. Laminins form a template for angiogenesis and facilitate astrocyte migration, as well as astrocyte–microglia communication [37,38]. Astrocytes are also able to secrete a spectrum of cell adhesion molecules (CAMs), contributing to synapse assembly, their functional specifications, and plasticity. Astrocyte-derived CAMs (reviewed extensively by Hillen et al., 2018 [39]) include, among others, the following factors: neuroligin, neurexin, cadherins, contactin, connexins, thrombospondin, and chondroitin sulfate proteoglycans. These compounds play key roles during neurodevelopment, like facilitating cell migration and process extension, as well as promoting nerve growth and synapse formation, as well as stabilization (while inhibiting axonal growth in the adult CNS after injury) [40,41,42,43,44,45,46]. Other well-recognized factors synthesized by astrocytes and involved in synaptogenesis are D-serine, TNF-α (tumor necrosis factor α), and TGF-β1 (transforming growth factor β1), which also act in an autocrine manner to regulate astrogenesis [47,48]. It is widely known that the control of synapse number and function is critical to the formation of neural circuits. Likewise, the number of synapses in direct contact with one astrocyte resulting from complex neurodevelopmental processes was estimated to be between 20,000 to 100,000 in rodents and up to 2 million in humans [49,50].

Among the major compounds released by astrocytes during CNS development, two crucial factors that promote neurogenesis can be distinguished: gliogenesis and conferring trophic support; these factors correspond to brain-derived neurotrophic factor (BDNF) and insulin-like growth factor 1 (IGF-1) [51,52,53]. Locally secreted by glial cells, IGF-1 is one of the major factors regulating the differentiation of oligodendrocytes into mature cells and supporting the branching of their processes, which are capable of axon myelination [54].

The neurodevelopmental processes that peak in the neonatal and early postnatal periods are well-orchestrated by a plethora of signaling and structural molecules. Astrocytes are recognized as key donors of the above-mentioned active compounds that govern crucial physiological developmental processes. However, what happens when pathophysiological conditions trigger astrocyte reactivity during this exceptionally sensitive period of ontogenesis?

## 3. Astrocyte Reactivity in Neurodevelopmental Disorders

Among the pathophysiological events affecting the fragile developing CNS, perinatal asphyxia is one of the leading causes of subsequent brain injuries. According to statistical data, 1–8 babies per 1000 live births experience fatal consequences of perinatal asphyxia. These data are considered to underestimate the real numbers due to poor medical care standards and a lack of precise reports from many developing countries [55,56]. A temporary reduction in blood circulation and the resulting limitations in oxygen and trophic supply trigger a cascade of intracellular biochemical changes. Temporal limitations in oxygen supply first lead to the inhibition of oxidative phosphorylation due to the rapid depletion of ATP reserves and lactic acid accumulation, resulting in metabolic acidosis, the release of excitatory neurotransmitters (especially glutamate, Glu), the dramatically increased production of free radicals, and the initiation of apoptotic cascades resulting in neuronal cell death. The resulting hypoxic–ischemic injury to the nervous tissue activates the cell response to the pathophysiological changes occurring in the local microenvironment, thus affecting the selected signal transduction pathways and resulting in the changed expressions of the active compounds. Accordingly, an increase in glutamate transporter proteins to re-uptake the glutamate, reduce its extracellular levels, and prevent its cytotoxicity is among the first cell responses to the microenvironmental changes evoked by hypoxia hypoxia–ischemia. Due to the elevated expression of glutamine synthetase, glutamate is intracellularly converted into glutamine and stored in the cellular vesicles. Enhanced superoxide dismutase activity helps clear the affected tissue from excess free radicals [57]. However, recent data from several laboratories suggest that these mechanisms become inefficient in later phases after insult and that the degree of astrocytic malfunction may be an indicator of the outcome following hypoxic and hypoxic–ischemic brain injury [58]. To support neighboring neural cells, the cell secretome changes in a microenvironmental-context-specific manner, further modifying the composition of the extracellular milieu [59,60,61]. Anti-inflammatory interleukins, predominantly IL-10, and a spectrum of chemokines help to overcome local neuroinflammation, making the local tissue microenvironment conducive to neuroregenerative processes [62,63]. Neurotrophins (NT3, BDNF) promote neurogenesis and exert a neuroprotective effect for the neurons that survived injury [52,64,65]. Increased levels of astrocyte-derived IGF-1, platelet-derived growth factor AA (PDGF-AA), and leukemia inhibitory factor (LIF) promote oligodendrocyte proliferation and differentiation [54,66,67,68,69,70]. Sonic hedgehogs released locally by activated astrocytes contribute to BBB recovery and stabilization via patched-1 (Ptch-1), which is an SHH receptor localized on BBB-forming cells. Suppression of PTch-1 by SHH binding activates transcription factor glioma-associated oncogene homolog-1 (Gli-1), regulating the expression of genes in endothelial cells involved in tight junction (TJ) formation [60,67,71].

Neonatal astrocytes are reported to be relatively resistant to oxygen and glucose deficiency. However, their activation affects intercellular communication with the cells of the developing CNS. The high significance of astrocyte–microglia and astrocyte–oligodendrocyte interplay has been shown in both neurodevelopmental processes and the modulation of cell responses to pathophysiological conditions, including neuroinflammation. While microglia populate the developing CNS during mid-embryogenesis, intense oligogliogenesis takes place soon after the peak of astrogenesis during neurodevelopment and is most pronounced in the early postnatal period. In rodents, the most intense period of oligogliogenesis occurs for 1–10 postnatal days, while in humans, it continues from mid-to-late gestation through the first postnatal weeks [72,73,74], although the process of CNS myelination is most active until the end of the 4th year, strongly depending on the brain region [75,76]. During this period, the majority of cells (at least oligodendrocytes) are in their precursor state, characterized by high susceptibility to extracellular clues, which governs their survival, proliferation, and differentiation. This makes precursors vulnerable to the harmful influence of pathophysiological conditions and is the primary reason for the high susceptibility of neonatal CNS to damage. The multidirectional responses of astrocytes to alterations in the composition of the local extracellular milieu, the presence of pathogens or experienced injury, and their interactions with neighboring cells in the nervous tissue seem to be crucial to initiate the mechanisms leading to tissue recovery or, conversely, foster neurodegenerative processes.

Many frequent perinatal disorders and injuries, such as obstructed labor, preeclampsia, perinatal asphyxia, intraventricular hemorrhage, neonatal stroke, and traumatic brain injury (TBI), are associated with a temporarily limited supply of oxygen and glucose, and astrocytes are recognized as functional oxygen sensors [77,78,79]. One of the major intracellular pathways that readily responds to tissue hypoxia is under the control of hypoxia inducible factors (HIFs), which act as intracellular oxygen sensors [80]. HIF-1 and HIF-2 are heterodimeric transcription factors consisting of HIF-1α and HIF-1β and HIF-2α and HIF-1β, respectively. The α-subunits act as the prime transcription factors, and the β-subunits (alternatively named ARNTs) help the α-subunits bind to DNA. Under physiological conditions, the HIF-1β protein subunit is constitutively expressed in the cell nuclei and translated at a low level, allowing to control the selected gene expression in the developing CNS and during physiological cell functioning. Depending on the brain region, these conditions correspond to approximately 5% oxygen [81] and, therefore, are commonly referred to as physiological normoxia. Conversely, HIF-1α is ubiquitinated and degraded by prolyl-hydroxylases under normoxic conditions, while stabilization of this subunit strongly depends on oxygen tension. As the defined concentration of molecular oxygen is pivotal to sustaining intracellular bioenergetics, HIFs as oxygen sensors control the activity of genes responsible for survival/apoptosis, pH regulation, mitochondrial turnover, and other biological processes crucial for cell biology. However, HIFα turnover is very rapid, with an intracellular half-life determined to be below 5 min, followed by proteasome-mediated degradation [82].

Under hypoxic conditions (corresponding to 0.5–2% O_2_, while O_2_ < 0.5% is recognized as anoxia), HIFs are stabilized, and their transcriptional activity affects several genes responsible for, e.g., glycolysis, erythropoiesis, and angiogenesis, which are essential for the neuroregeneration of neonatal brains injured by a cascade of biochemical reactions triggered by transient hypoxia–ischemia. HIF-1α stabilization and enhanced transcriptional activity are also observed during inflammation when microglia cells are activated, and the nervous tissue is invaded by oxygen-consuming immune cells. The higher energy demands by active immune responses to pathogens contribute to a decrease in available oxygen concentrations and thus affect HIFs as oxygen sensors. These factors were, however, shown to be directly activated by signaling factors engaged in immune responses, such as pro-inflammatory cytokines and bacterial lipopolysaccharides [83,84,85], predominantly via the canonical NF-κB signaling pathway (nuclear factor kappa-light-chain-enhancer of activated B cells signaling pathway) [86]. Neuroinflammation and the upregulated production of cytokines are evoked in neonatal brains not only due to infections but also as a result of perinatal asphyxia [87].

Likewise, the main intracellular target of activated HIFs is erythropoietin (EPO). This hematopoietic glycoprotein hormone regulates the expansion of erythroid progenitor cells by inhibiting their apoptosis and promoting their differentiation into red blood cells [88]. In this way, EPO secures the oxygen capacity of the blood and its supply to the body’s organs [89]. EPO, however, was also shown to be expressed in the neural cells of the developing brain, including astrocytes. Moreover, an astrocyte-derived EPO was identified as a key mediator of paracrine neuroprotection under hypoxic conditions [90,91]. In cultured cortical astrocytes activated by hypoxia (1% O_2_), the expression of EPO was significantly increased and attenuated neuronal damage [92,93]. As shown by several studies, the neuroprotective properties of EPO are conferred by activating endogenous antioxidant, antiapoptotic, and anti-inflammatory signaling pathways (reviewed by, e.g., Vittori, 2021 [94]).

Another target of HIF activity in reactive neonatal astrocytes that contributes to enhancing endogenous restorative mechanisms is VEGF, the aforementioned factor that stimulates the formation of new blood vessels to compensate for oxygen deficiencies in the affected nervous tissue. However, as demonstrated by numerous in vitro and in vivo studies, VEGF can promote neurogenesis [95,96,97,98], directly supporting tissue repair. In this context, the induced astrocytic reactivity associated with HIF activation is beneficial for neuroregeneration.

Taken together, HIFs are targeted by numerous types of incidents associated with a temporarily limited supply of oxygen, thereby affecting the developing CNS. The upregulated transcriptional activity of HIFs regulates the expression of genes engaged in several metabolic processes (e.g., glucose transporter-1 (GLUT1), pyruvate dehydrogenase kinase 1 (PDK1), and lactate dehydrogenase A (LDHA)). Since astrocytes contribute to supplying oxygen and energy substrates for highly demanded cells such as neurons and oligodendrocytes (since the transduction of nervous signals and myelin elaboration are intensely energy-consuming processes), the activation of astrocytic HIFs and subsequent upregulation of cell metabolism in response to oxygen and glucose restriction help guarantee continuous cell functioning in the altered microenvironment [99]. Thus, clinical strategies based on pharmacological modulation (e.g., via the prolyl hydroxylase inhibitor PHI FG-4497, [100]) of the evoked activity of HIFs might protect the affected tissue by helping to sustain cell metabolism and fostering tissue restoration through enhanced angiogenesis, erythropoiesis, and cell proliferation.

## 4. Impact of Glial Cell Crosstalk on Their Reactivity

**Figure 2:**
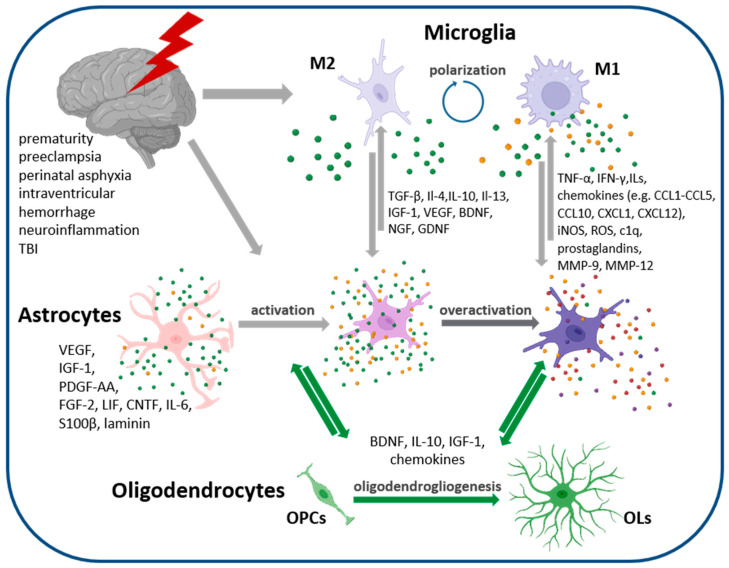
Cellular interaction of neonatal neural cells. Gliogenesis peaks in the perinatal period and generated astrocytes are very sensitive to instructive signals and clues present in the extracellular milieu, as well as those released by neighboring cells in the nervous tissue. Pathophysiological conditions or injuries trigger cell responses and changes in their secretome, influencing other cells constituting the developing nervous tissue in a paracrine manner. The activation of astroglia, recognized as astrogliosis, seems to facilitate neurorestorative processes, while astrocyte overactivation is thought to exert detrimental effects on tissue regenerative capacity. Abb.: M1 pro-inflammatory microglia phenotype, M2-anty-inflammatory microglia phenotype, OPC–oligodendrocyte progenitor cells, OL–oligodendrocytes. Figure created with BioRender.com (accessed on 17 November 2023).

Glial cells were shown to readily react to pathological clues and pathophysiological conditions affecting nervous tissue (Figure 2). Microglia are considered to be the most attentive CNS guardians, surveying the surrounding tissue microenvironment. Microglia respond to changes in the extracellular milieu composition or the presence of exogenous invaders by changing their resting phenotype; they also initiate a series of intracellular processes to combat exogenous threats and restore local tissue homeostasis. Depending on the cell morphology and profile of secreted chemokines and cytokines, two major microglial phenotypes are recognized: pro-inflammatory (named M1) or anti-inflammatory (known as M2). The main processes underlying the cellular responses of microglial cells include the reorganization of the microglial secretome in the context of releasing signaling molecules and, if necessary, changing cell morphology to an amoeboid form associated with phagocytic activity. Eliminating pathogens or cell debris by phagocytizing microglia helps overcome local neuroinflammatory processes and allows the initiation of endogenous restorative mechanisms. Additionally, secreting activity allows crosstalk with neighboring cells in a paracrine manner to modulate in situ processes.

Microglia, representing between 5% and 10% of the total cell number of the brain [23,101], release signaling molecules, including a plethora of cytokines, chemokines, and growth factors. Interestingly, microglia are themselves a source and a target of cytokines, influencing their polarization. Likewise, interleukin-4 (IL-4), interleukin-10 (IL-10), and interleukin-13 (IL-13) are able to induce the M2 microglial phenotype (in which three additional subtypes as additionally distinguished), whereas in the presence of TNF-α, interferon-γ (IFN-γ), or interleukin-1β (IL-1β), microglia predominantly acquire the M1 phenotype [102,103,104,105]. Likewise, the factors secreted by microglia exert either pro-inflammatory or anti-inflammatory effects. The former are conveyed by secreted factors like TNF-α, interleukins (IL-1β, IL-6, IL-17, Il-18, Il-23), numerous chemokines (including CCL5, CCL20, CXCL1, CXCL9, and CXCL10 acting as chemoattractants to recruit immune cells), inducible nitric oxide synthase (iNOS), reactive oxygen species (ROS), the complement component c1q, and prostaglandins. The latter beneficial anti-inflammatory processes, conducive to the initiation of endogenous compensative mechanisms in developing brains, are promoted by the factors released via the M2 phenotype and include TGF-β, IL-10, IGF-1, glial-derived neurotrophic factor (GDNF), BDNF, chemokines CCL13, CCL14, CCL17, CCL18, CCL22, and many others [106,107]. The microglia-derived factors modulate the cell responses of the astrocytes, which themselves are activated under pathophysiological conditions [61,108]. And conversely, astrocyte reactivity stimulates microglial responses, as shown in many in vitro and in vivo studies [109,110,111].

Although astrocyte and microglia reactivity are thought to be the key players in resolving between neurodegeneration and neuroprotection, there is, however, another neural cell type that easily responds to the imbalance of tissue homeostasis and pathological clues occurring in the CNS. Oligodendrocytes, which belong to macroglia along with astrocytes, exhibit strong immunomodulatory properties and confer trophic and protective support to the neighboring cells of the nervous tissue by releasing IL-10, BDNF, and IGF-1, among other active molecules [54,112,113,114]. Accordingly, the resulting degree of astrocyte reactivity is not only shaped by the pathophysiological conditions or clues present in the extracellular milieu but also modulated by the active molecules released by cross-talking cells of the nervous tissue.

Building on the evidence from numerous studies, especially those performed on the process of myelinogenesis/demyelination, it is currently presumed that the state of astrocyte reactivity either supports neuroregeneration or contributes to the progress of neurodegeneration. In the former “mild” state of activation, astrocytes confer trophic support by releasing neuroprotective molecules (especially BDNF), participate in the restoration of the BBB, regulate blood flow, and are engaged in the restoration of homeostatic balance [115]. Mitogen FGF-2 (fibroblast growth factor 2) secreted by astrocytes promotes neurogenesis from neural stem cells, supporting endogenous neurorestorative mechanisms in the affected nervous tissue. Simultaneously, VEGF stimulates angiogenesis, providing diseased tissue with oxygen and metabolic substrates, which are indispensable for enhanced biological processes of neurorestoration. In the case of dys/hypomyelination, astrocyte-derived active molecules like IL-6, CNTF, and LIF stimulate oligodendrocyte progenitor cells (OPCs) survival and proliferation and promote their differentiation into mature oligodendrocytes capable of myelinogenesis [116]. This phenomenon is of special importance in neonatal diseases since the prenatal and early postnatal periods coincide with intense OPC generation and developing brain myelination. OPCs are extremely sensitive to the influence of extracellular clues, including even the short-term deprivation of oxygen and glucose, which strongly affects their survival and hampers their maturation. As a consequence, the process of myelinogenesis is seriously altered, resulting in deficient myelination or aberrations of the myelin sheaths elaborated by the affected oligodendrocytes. Astrocyte reactivity, which is triggered by the same clues, as in the case of oligodendrocytes, not only supports the restoration of local tissue homeostasis but can also help to rescue OPCs and promote their maturation [117].

## 5. Neurorepair versus Neurodegeneration

### 5.1. Neonatal Disorders: Reactive Astrogliosis in the Selected Neonatal Diseases

In the context of the immunomodulation of processes evoked in cellular responses to neonatal disorders, the stimulation of astrocyte reactivity could be beneficial for combating pathophysiological clues and for tissue recovery. The major threat to neuroregeneration seems to be associated with astrocyte overactivity, which leads to the biosynthesis of pro-inflammatory cytokines, the secretion of contact molecules, and the generation of high levels of NO (nitric oxide). The local tissue microenvironment is modulated by astrocyte-derived compounds, and its composition hampers the initiation of endogenous reparative processes. Governing the state of astrocyte activation can be a target of potential therapeutic strategies for neonatal brain disorders.

Astrocyte overreactivity and astrogliosis are common features of numerous neonatal and pediatric diseases. One of the best-described disorders is the follow-up to pathophysiological events evoked by neonatal hypoxia–ischemia. Perinatal asphyxia, due to a temporary limited supply of oxygen and nutrients, leads to the cellular responses of neural cells. As shown by the in vitro studies, even a short episode of oxygen and glucose deprivation triggers the proliferation of neonatal astrocytes and yields changes in their secretory activity [23,54]. The hypertrophy of astrocytes together with an increase in the expression of GFAP culminating in the formation of glial scars in the damaged nervous tissue of neonatal brains was also observed in an in vivo animal (rat and sheep) model of perinatal hypoxia–ischemia [118,119] and an ischemia model in near-term fetal sheep [120]. Interestingly, not only a shortage of trophic support but also overnutrition in the postnatal period was postulated to trigger hypothalamus astrogliosis in rat models [121]. Astrogliosis is also a common feature of other neonatal disease conditions like intraventricular hemorrhages in pre-term children [122], neuroinflammation evoked by sepsis [123], hyperbilirubinemia [124], and other intrauterine and postnatal infections (especially in preterm born children), including coronavirus disease 2019 (COVID-19) inflammation [125,126]. In recent studies, SARS-CoV-2 was shown to exhibit tropism towards human cortical astrocytes [127,128]. Astrocyte overreactivity was also observed as a consequence of neonatal TBI, where the resulting epilepsy was shown to be associated with subacute hippocampal astrogliosis, which was also present in the neocortex [129,130]. However, additional factors beyond pathophysiological conditions or injury can lead to astrocyte activation. As shown in studies on non-human primates, intentionally applied medical conditions, like anesthesia, trigger increased astrogliosis in selected brain regions (predominantly in the amygdala) and persistent astrocyte reactivity, which correlates with social behavioral deficits [131]. Other studies reported compromised astrocyte morphogenesis after anesthesia in neonatal mice [132]. In the context of data from several studies on the activation of neonatal astrocytes, a question arises about the possibility of effectively governing the state of their reactivity to promote potentially beneficial effects for the restoration of physiological functions in developing nervous tissue.

As mentioned in the previous sections, the perinatal and postnatal periods are the stages of neurodevelopment in which processes of gliogenesis proceed most intensely. Newly generated astrocytes populate the developing nervous system and are very sensitive to the influence of external clues that govern their migration and the role(s) they play in that particular period of nervous system formation. Imbalances in local tissue homeostasis, such as oxygen and nutrient deficiency, can trigger the cellular responses of the cells inhabiting nervous tissue. Astrogliosis is usually one of the first symptoms of neuroinflammation, in which the microglia become polarized and contribute to in situ on-going processes by secreting factors modulating the composition of the extracellular milieu. Alterations in the tissue microenvironment exert effects on extremely sensitive OPCs, which are also intensely generated during this particular period of neurodevelopment. Thus, the multidirectional crosstalk between neural cells might be the discriminating factor between neurorepair and neurodegeneration. Maintaining astrocyte reactivity in a “mild state” of activation seems to be one of the most important goals in combating the development of pathophysiological mechanisms initiated by various factors. Accordingly, astrocytes are well known to sense extrinsic signals, which trigger their reaction, which is referred to as astrogliosis. Depending on the degree of cell activation and changes in the secretome, composition, a gradient in cell reactivity can be observed in the ongoing process, ranging from mild to moderate, then from moderate to severe, and finally to the formation of a glial scar [61,133,134]. There is a consensus that mild to moderate astrogliosis is associated with essential beneficial functions, whereas astrocyte overreactivity usually exerts harmful effects. Similar to the very simplified nomenclature of reactive microglia (i.e., M1 versus M2 phenotype), the astrocyte phenotypes associated with either mild or moderate astrogliosis were named A2, whereas overactivated phenotypes were recognized as A1. The former phenotype was shown to confer neurotrophic and pro-regenerative support by upregulating the secretion of a plethora of beneficial active factors, including, among others, BDNF, nerve growth factor (NGF), GDNF, CNTF, LIF, PDGF-AA, (platelet-derived growth factor-BB) PDGF-BB, and FGF [135,136]. Regardless of their nomenclature, which remains a debatable issue [137], the modulation of astrocyte reactivity by enhancing their protective features seems to be one of the most promising strategies for diseased nervous tissue.

### 5.2. Crosstalk between Neural Cells in the Developing Brain

The secretory activity of astrocytes reacting to alterations in local homeostasis evoked by pathophysiological clues contributes to further modulation of the composition of the extracellular milieu. The active compounds released into extracellular compartments in varying concentrations influence the functioning of the cells populating and inhabiting the developing brain. As mentioned in the previous sections, microglia are the primary caretakers of CNS functioning, rapidly reacting to pathophysiological clues in the local tissue microenvironment. In response, microglia can release a plethora of factors acting like instructive signals in a paracrine manner [138,139,140,141]. These microglia-derived signals can include (depending on the type of occurring pathology) cytokine TNF-α and interleukins (like IL-1β, IL-6, IL-12, IL-17, IL-18, and IL-23), chemokines (including CCL1-CCL5, CCL10, CXCL1, and CXCL12), complement component C1q, prostaglandins, NO, and ROS, which stimulate astrocyte reactivity, presumably via the nuclear translocation of NF-κB, whose central role in astrocyte activation was demonstrated in many neural disorders [142,143,144]. In this way, astrocytes become overreactive, contributing to the enhancement of neuroinflammatory processes and degeneration.

Astrocytes were also shown to interact with other types of macroglia such as oligodendrocytes, whose immunomodulatory and neuroprotective features were demonstrated in numerous studies [112,113,145,146,147]. Accordingly, oligodendrocytes and their progenitors release pro-neuroregenerative BDNF and numerous cytokines, including anti-inflammatory IL-10, thus attenuating astrocyte activity. However, the stressed cells could be the sources of IL-1β and IL-17 or CXCL10 and CCL2, which are able to exacerbate inflammation. In response, astrocytes up-regulate secretion of the selected factors (e.g., IGF-1, PDGF, FGF-2, LIF, CNTF, IL-6, S100 calcium-binding protein B–S100β, and laminin) affecting oligodendrocytes in a context-dependent manner, leading to hypomyelination, which is often accompanied by malformation of the elaborated myelin sheaths [54,148,149,150,151].

Altogether, the resulting astrocyte activity is influenced not only by pathophysiological clues but also by the resulting cellular paracrine communication between affected neural cells. Intercellular crosstalk is especially important during neurodevelopment when cells are particularly sensitive to the influence of the instructive signals governing their migration, survival, commitment, and differentiation. On the one hand, this fragility opens highly desirable opportunities for natural endogenous neuroregenerative mechanisms. On the other hand, this phenomenon makes the cells exceptionally vulnerable to the influence of a broad spectrum of compounds, even in relatively low concentrations, triggering their overreactivity or leading to necrosis/apoptosis.

In this context, the regulation of astrocyte reactivity (mild, anti-inflammatory, and pro-regenerative versus strong reactivity, thereby contributing to the exacerbation of neuroinflammation and neurodegeneration) might be achieved by modulating the activation of other cell types through an etiology-based approach. Anti-inflammatory treatments are one of the first-line therapies for microglia reactivity. Depending on the type of pathogen or insult, pharmacological therapy can be based on the application of antibiotics, factors influencing M1 to M2 phenotype polarization (IL-4, IL-13, and TGF-β); analogs of physiological substances (melatonin, erythropoietin, omega-3 polyunsaturated fatty acids, and many others); or natural, usually plant-derived substances (like, e.g., resveratrol, curcumin) [152,153,154,155,156,157,158]. Enhancing oligogliogenesis, resulting in an increased number of OPCs conferring neurotrophic and immunomodulatory support, might be another way to modulate astrocyte reactivity. This enhancement could be achieved through the application of PDGF-AA, a physiological mitogen that plays a crucial role in OPC commitment and proliferation. The excess of generated OPCs is eliminated during neurodevelopment. Thus, focusing on OPCs as natural helpers of neuroregeneration could be tested as a potential strategy. In the context of glial scar formation, it should be considered that OPCs largely contribute to their structure. Thus, such a strategy is not recommended for preventing the results of acute injuries.

Among the most promising strategies for modulating astrocyte overreactivity, those based on the application of stem cells, especially those derived from the umbilical cord, could be distinguished, and taken into account to develop strategies for neonatal and pediatric diseases. The regenerative potential of stem cells is recognized today as offering broad, unique paracrine support for diseased tissue [159,160,161,162]. Most notably, the umbilical cord, which is usually discarded after labor, is considered one of the best sources of stem cells for clinical purposes. These cells are characterized by low immunogenicity and many beneficial active compounds (including neurotrophic and pro-angiogenic factors) compared to the cells derived from other sources [163,164,165]. Thus, the stromal/mesenchymal stem cells derived from human umbilical cord blood and Wharton’s jelly are able to confer anti-inflammatory and pro-regenerative effects (like CCL2, hepatocyte growth factor—HGF, VEGF, BDNF, NGF, FGF-2, and stromal cell-derived factor 1α—SDF-1α), thereby modulating astrocyte reactivity and providing neurotrophic support [166,167]. Moreover, stem-cell-based therapies were shown to be effective when applied in the form of cell-free protocols using the administration of stem-cell-derived microvesicles, especially exosomes carrying physiological therapeutic factors [32,168]. The extracellular vesicles used exclusively as clinical treatments for the modulation of neonatal astrogliosis could be manufactured as off-the-shelf medicine, also facilitating their application outside the neonatal intensive care unit (NICU). To date, the promising effectiveness of stromal/mesenchymal stem cell transplantation in governing neonatal astrogliosis has been demonstrated by many studies dedicated to perinatal asphyxia, intraventricular hemorrhages, and infections [169,170,171,172,173].

## 6. Conclusions

In conclusion, astrocytes are easily activated during brain development, especially in the perinatal period. Their reactivity affects cells populating developing nervous tissue, leading to abnormalities and, in the most severe cases, long-lasting neurodisabilities. Taking into consideration the significant vulnerability of astrocytes and other types of neural cells to the influence of external clues in the fragile developing brain, one target of potential strategies is to treat the evoked astrocyte reactivity as a friend instead of a foe for the regeneration of the brain affected by pathogens or insults.
